# Characterization of Exoelectrogenic Bacteria *Enterobacter* Strains Isolated from a Microbial Fuel Cell Exposed to Copper Shock Load

**DOI:** 10.1371/journal.pone.0113379

**Published:** 2014-11-20

**Authors:** Cuijie Feng, Jiangwei Li, Dan Qin, Lixiang Chen, Feng Zhao, Shaohua Chen, Hongbo Hu, Chang-Ping Yu

**Affiliations:** 1 Institute of Urban Environment, Chinese Academy of Sciences, Xiamen, 361021, China; 2 Ningbo Urban Environment Observation and Research Station-NUEORS, Chinese Academy of Sciences, Ningbo, 351800, China; 3 State Key Laboratory of Microbial Metabolism, Shanghai Jiao Tong University, Shanghai, 200240, China; Texas A&M University, United States of America

## Abstract

Microorganisms capable of generating electricity in microbial fuel cells (MFCs) have gained increasing interest. Here fourteen exoelectrogenic bacterial strains were isolated from the anodic biofilm in an MFC before and after copper (Cu) shock load by Hungate roll-tube technique with solid ferric (III) oxide as an electron acceptor and acetate as an electron donor. Phylogenetic analysis of the 16S rRNA gene sequences revealed that they were all closely related to *Enterobacter ludwigii* DSM 16688^T^ within the Enterobacteriaceae family, although these isolated bacteria showed slightly different morphology before and after Cu shock load. Two representative strains R2B1 (before Cu shock load) and B4B2 (after Cu shock load) were chosen for further analysis. B4B2 is resistant to 200 mg L^−1^ of Cu(II) while R2B1 is not, which indicated the potential selection of the Cu shock load. Raman analysis revealed that both R2B1 and B4B2 contained c-type cytochromes. Cyclic voltammetry measurements revealed that strain R2B1 had the capacity to transfer electrons to electrodes. The experimental results demonstrated that strain R2B1 was capable of utilizing a wide range of substrates, including Luria-Bertani (LB) broth, cellulose, acetate, citrate, glucose, sucrose, glycerol and lactose to generate electricity, with the highest current density of 440 mA·m^−2^ generated from LB-fed MFC. Further experiments indicated that the bacterial cell density had potential correlation with the current density.

## Introduction

In a microbial fuel cell (MFC), electroactive microorganisms are capable of generating electricity directly from organic compounds. Due to their specific ability to transfer electrons outside the cell to the anode of the MFC, these bacteria are renowned as exoelectrogens (“exo-” for extracellular and “electrogens” for the ability) [1]. The performance and power capability of MFCs also depend on the kinetics of the electron transfer. Studies on the mechanisms for extracellular transport of electrons by different bacteria are still underway. Electron transfer mechanisms can be generally divided into direct electron transfer (DET) and mediated electron transfer (MET). DET can take place directly via membrane bound cytochromes [2]–[3] or via electrically conductive nanowires (pili) [4]–[6]. For the MET, the electron transfer can be mediated by redox mediators [2], [7] or oxidation of reduced secondary metabolites (Plavins, Phenazine, etc.) [7]–[9].

It is obvious that exoelectrogens play a pivotal role in the process of electron transfer from cell to electrode. Currently, different pure isolates have been reported in the literature as exoelectrogens, such as *Geobacter* sp., *Shewanella* sp., *Pseudomonas* sp., *Burkholderia* sp., *Escherichia coli*, *Ochrobactrum anthropi, Rhodoferax ferrireducens*, *Citrobacter* sp. and *Clostridium sticklandii* (Table S1). Microbial reduction of Fe (III) is an important process of anaerobic redox cycling of iron as well as degradation of natural or contaminant organics in different environments, such as freshwater, sediments and aquifers [10]. In previous studies, a series of iron oxides, including ferric citrate, Akaganeite (β-FeOOH), Goethite (α-FeOOH) and Fe(III)-pyrophosphate were used for the isolation of exoelectrogens [11]. This is because many isolated iron-reducing bacteria were shown to display electricity generating ability when applied to MFC system. For example, iron-reducing bacterium *Geobacter sulfurreducens* strain PCA within the δ-Proteobacteria was initially isolated from a petroleum-contaminated shallow aquifer [11]–[12], and the maximum power density in MFC obtained from *G. sulfurreducens* strain PCA was 1.88 W m^−2^ by oxidizing acetate [13]. A continuous effort is undertaken to explore new exoelectrogens which have the capacity to transfer electrons to electrodes as well as to degrade specific contaminants. For instance, Rezaei et al. [14] isolated a new strain similar to *Enterobacter cloacae*, which could achieve simultaneous cellulose degradation and electricity generation with a power density of 4.9±0.01 mW m^−2^.

Wastewater composition could contain potential toxicants such as heavy metals. Sudden surges of high concentration of metals could deteriorate the performance of the biological treatment process. In view of the potential risks of heavy metals in various wastewater steams [15]–[16], developing new quick-responding and cost-effective detection system are necessary. The MFC systems have the potential to detect the toxic metals in the influent as the inhibition effects can be reflected directly by the variation of voltage data online [17]–[19]. Meanwhile, sudden shock load of toxic metals could lead to the changes of microbial population in MFCs. In particular, exoelectrogens in the anode biofilm may be affected due to the MFC's exposure to high concentration of toxic heavy metals and these changes are still unclear.

In our previous study, a continuous flow MFC system was operated for decentralized wastewater treatment, and experiments were also conducted to test the MFC response to a shock load of copper (Cu) [19]. The results showed that although the shock load of Cu would completely inhibit the electricity production, the microbial populations restored their ability to produce electricity afterwards. In this study, we report the isolation of bacteria capable of producing current from the previous MFC system before and after Cu shock load. Also, the biochemical, physiological, and electrochemical characteristics of two representative strains were further characterized.

## Materials and Methods

### Isolation and biochemical characteristics of isolated exoelectrogens

A continuous-flow MFC was constructed to contain three rectangular chambers (anodic compartment, cathodic compartment and internal clarifier), which were separated by Plexiglas baffle without conventional proton exchange membrane (PEM). [19]. Two holes with a diameter of 5 mm were made on the baffle between the anodic and cathodic compartments to allow wastewater to flow though the system. The MFC was inoculated with anaerobically enriched activated sludge and fed with the synthetic wastewater with sodium acetate as the carbon source as previously described [19]. Carbon cloth (10×10 cm) was used as the electrodes for both the anode and cathode, except the cathode carbon cloth was brush-coated with the FePc catalyst [20]. Exoelectrogens were isolated from the anode biofilm of the MFC operated over a period of five months without Cu(II) shock load. After the MFC was exposed to the Cu(II) shock load and recovered its ability to produce electricity (210 d∼), exoelectrogens were isolated from the anode biofilm again. Cells were extracted by vortexing a portion of carbon cloth anode (4 cm^2^) in the anaerobic serum bottle (100 ml) containing 50 ml of sterile anaerobic culture medium. The isolation was performed by serial dilution using Hungate roll-tube technique with the basal medium (BM) [21]. The BM contained 0.115 g NH_4_Cl, 0.026 g KH_2_PO_4_, 1.64 g sodium acetate, 0.2 g yeast extract (Oxoid), 0.5 g cysteine hydrochloride, 1 mg resazurin (Sigma), 12.5 ml mineral and 12.5 ml vitamin solutions per liter (Lovley and Phillips, 1988). The electron acceptor was 20 mM amorphous Fe(III) oxyhydroxide, which was synthesized by neutralizing a 0.4 M FeCl_3_ solution to a pH of 7 with 1.2 M NaOH [22]. The medium was boiled under a stream of O_2_-free N_2_ gas and cooled to room temperature. Five-mililiter of the medium was dispensed into Hungate tubes and 50 ml medium was dispensed into serum bottles. The sealed vessels were then autoclaved for 20 min at 121°C. The culture was purified by using a repeated Hungate roll-tube method with BM agar. Single black colonies were picked out and inoculated into the same liquid medium.

Substrate utilization of isolated strains was determined by conventional methods using Biolog GN2 MicroPlates (Biolog, Inc., Hayward, CA). Enzyme activity was carried out on API ZYM (bioMérieux) according to the manufacturer's instructions.

### 16S rRNA gene sequencing and phylogenetic analysis

To confirm the purity and the similarity of the isolated strains, genomic DNA was extracted using the FastDNA SPIN Kit for Soil (Qbiogene-MP Biomedicals, Irvine, CA, USA) according to the manufacturer's instructions. The 16S rRNA gene was PCR amplified using the primer 27F (5′-AGAGTTTGATCMTGGCTCAG-3′) and 1492R (5′-GGTTACCTTTGTTACGACTT-3′) [23]. PCR solutions contained 25 µl Premix Ex Taq (TaKaRa Biotechnology Co., Ltd, Dalian, China), 1 µM of each primer, 0.5 µg of DNA template, and sterile deionized water to make up the total volume of 50 µl. PCR amplification was carried out in a Gene ProThermal Cycler (Bio ER, Hercules, China) with an initial denaturation of DNA for 5 min at 94°C, followed by 30 cycles of 30s at 94°C, 45 s at 55°C, and 1.5 min at 72°C, and then final extension for 7 min at 72°C. The 16S rRNA gene sequence obtained was compared to the sequences of the most closely related reference strains in the GenBank database via the BLAST program. A phylogenetic tree was constructed using the neighbor-joining algorithms (Saitou and Nei, 1987) with the Molecular Evolutionary Genetics Analysis software (MEGA 6) [24]. The 16S rRNA gene sequences have been registered in the GenBank, EMBL and DDBJ nucleotide database under the accession numbers: KJ956390- KJ956403.

### Procedures of copper-resistant experiments

A series of LB agar containing different Cu(II) concentrations, including 0, 50, 100, 150, 175, 200, 250, 300 to 400 mg L^−1^, was prepared for the copper-resistant experiment. Two representative isolated exoelectrogens, strain R2B1 and strain B4B2 were used in this study. A metal and antibiotic sensitive strain, *Escherichia coli* 5× RND (a gift from Professor Christopher Rensing) was also used for comparison in the assay [25].

### Electron microscopy

The morphologies of bacterial cells growing overnight in anaerobic tubes were examined with a scanning electron microscope (SEM) (S-4800, Hitachi Corp., Japan). For SEM, the suspended cells were fixed in a 2.5% glutaraldehyde and 0.1 M phosphate buffer solution (0.2 M, pH 7.2) for 2 h, and then dehydrated with a graded ethanol series from 30 to 100% for 20 min each. After dehydration, the samples were critical-point dried and then sputter-coated with gold under vacuum for SEM examination [26].

A 5 µl cell suspension was placed on 200 mesh Formvar carbon-coated copper grid and wicked off after 3 min. The sample was soaked in 5 µl of uranyl acetate (2%) for 30 s, then drained and air-dried and examined using a transmission electron microscopy (TEM) (H-7650, Hitachi Corp., Japan) at an accelerating voltage of 80 kV.

### Electrochemical analysis

Cyclic voltammetry (CV) was performed using a potentiostat (CHI 630D, CH Instruments Inc., CA) to investigate the electron transfer mechanism. The CV experiment was conducted with a three-electrode system with glassy carbon electrode as working electrode, Ag/AgCl as reference electrode, and a platinum wire as counter electrode according to a previous study [27]. The electrodes were cleaned in ethanol and rinsed with deionized water prior to use. The isolated bacteria were coated on a rounded surface (3 mm in diameter) of working electrode using Nafion ionomer (5% dispersion) as a binder. CV curves were determined in the potential range of -0.4 to +0.8 V vs. Ag/AgCl at a scan rate of 20 mV/s (CHI 630D, CH Instruments, USA). Phosphate buffer solution (PBS) (0.05 M, pH 7.2) was deoxygenated by purging with nitrogen gas for 30 min before and during the measurement. The strain R2B1 cells were cultured for 17 h and then washed for several times using PBS solution. The washed cells were also used for the chronoamperometric tests. To evaluate current generation, i-t experiment was conducted at a potential of 0.4 V vs. Ag/AgCl supplied with an electrochemical workstation (CHI 630D, CH Instruments, USA) by a three-electrode system. Twenty-microliter acetate (10 mM) was added into the PBS buffer to evaluate the current change. All the electrochemical experiments were conducted in triplicate.

### Confocal Raman microscopy analysis of electrochemically active microbial biofilms

To perform confocal Raman microscopy, the anodes were cut from the incubation vessels and transferred immediately into a sterile cell culture dish filled with 50 ml of PBS. Raman spectra were acquired using a confocal micro-Raman system (LabRAM Aramis, HORIBA Jobin Yvon, France) equipped with a Leica microscope, charge coupled device detector. Excitation was provided by a He-Ne 532 nm laser with the power of 5 mW on the sample. Raman signals were collected with a 50 mm optical fibre with a resolution of 4 cm^−1^. Spectral acquisition was done at integration times of 0.2 s to obtain high-contrast resonance spectra for the c-type cytochromes.

### Evaluation of power generation in MFCs

Two-chamber MFCs were constructed for evaluation of power generation by the isolated *Enterobacter* strain R2B1 using various substrates including citrate, acetate, glucose, sucrose, glycerol, lactose, cellulose and LB medium in a fed-batch mode in an incubator (30±2°C). Pure cellulose was microcrystalline insoluble cellulose, 15% amorphous and 85% crystalline with a 50-mm particle size (Sigmacell1, Sigma-Aldrich Co., St. Louis. MO). The MFCs were made of Plexiglas and anodic and cathodic compartments had an internal dimension of 5×5×5 cm, which were separated by a PEM (Zhengjiang Qianqiu, China). The PEM was pretreated by boiling it in 3% hydrogen peroxide for 1 h, and subsequently in 1.0 M sulfuric acid for another 1 h in boiling deionized water, and then stored in deionized water before use. Carbon felt with a surface of 3×3 cm was used as the electrode material. The anode solution (100 ml) was modified based on 0.05 M PBS (pH = 7.4) containing 0.12 g NH_4_Cl, 8 g NaCl, 0.13 g KCl, 7.8 g NaH_2_PO_4_·2H_2_O, 17.9 g Na_2_HPO_4_·12H_2_O, and 12.5 ml mineral and 12.5 ml vitamin solutions per liter [28]. For each substrate, at least eight batches were run to investigate current output at a fixed external resistance of 1 kΩ and under the same initial substrate concentration of 20 mM. PBS was used to maintain the solution pH and conductivity. The cathode solution (100 ml) consisted of 0.1 M potassium ferricyanide [K_3_Fe(CN)_6_] in PBS (pH = 7.0, 0.1 M). MFCs were inoculated with 3 ml late exponential phase cultures of isolated bacteria grown in the LB broth.

### Analysis and calculations

Cell voltage (V) was recorded online every 10 min via a data acquisition system (USB7660-B, ZTIC, China) which was connected to a personal computer. The current (I) was calculated from the fixed external resistance (R = 1 kΩ) and cell voltage (V) according to Ohm's law (I = V/R). Power (P) was calculated by using P (mW m^−2^)  = 10×V^2^/(R×A), where V (mV) is the output voltage, A (cm^2^) is the surface area of the anode, and R (Ω) is the external resistance [29]. The current obtained was normalized to the surface area (100 cm^2^) of the cathodic electrode.

## Results and Discussion

### Isolation and phylogenetic analysis of potential exoelectrogenic bacteria

As shown in Figure S1, the black colonies growing on the surface of the tube wall proved that the Fe(III) was reduced by electrochemically active microorganisms to ferrous iron [Fe(II)]. Black colonies were picked and subcultured for further purification. Ultimately, six isolates (strains R2B1, R2B1, R2B2, R2W, R1W, R1B2) were obtained from a long-term operated MFC before Cu shock load, while after Cu shock load, we obtained eight isolates (strains B5W, W1W1, W2B2, W1B1, B4B2, B2B2, B2B1 and B4B1).

**Figure 1:**
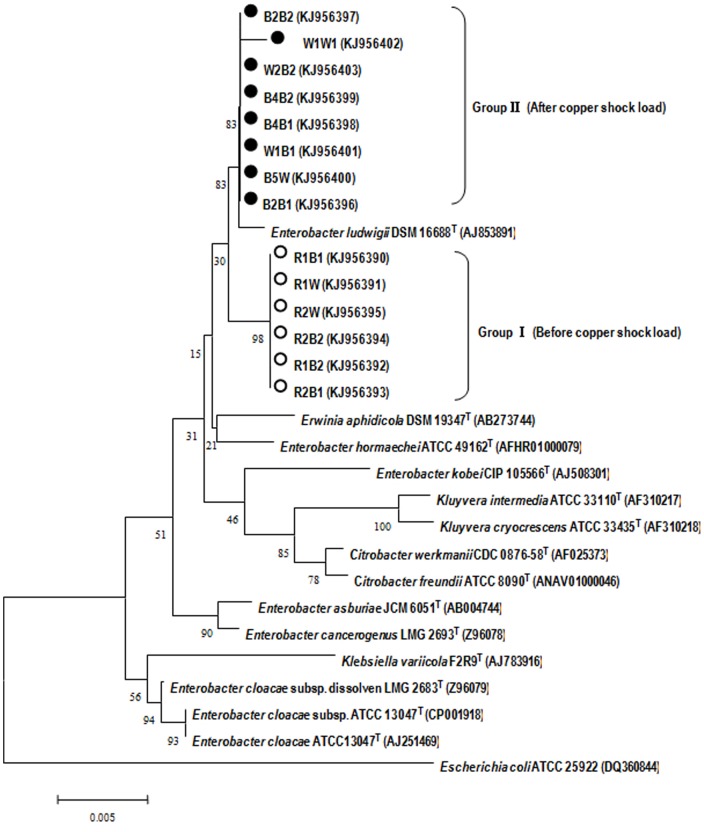
Phylogenic tree showing the relationships between isolated strains and closely related species based on 16S rRNA gene sequences. The sequence of *Escherichia coli* ATCC 25922 served as an outgroup sequence. The tree was constructed using the neighbor-joining method. The numbers at nodes indicate the percentages of occurrence of the branching order in 1000 bootstrapped trees. Scale bar  = 0.5% divergence.

**Figure 2:**
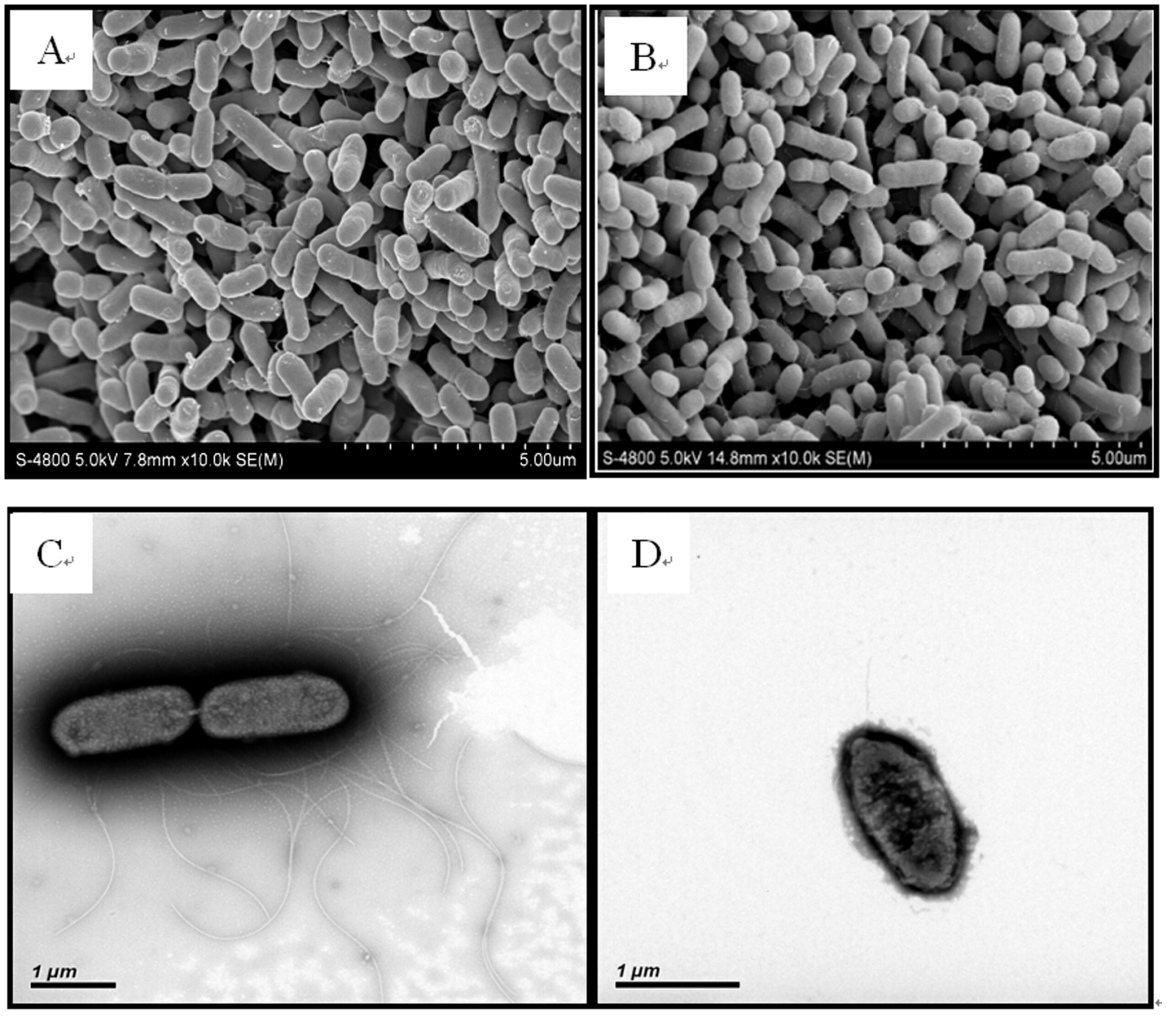
Micrographs of *Enterobacter* sp. strains R2B1 and B4B2. SEM of strains R2B1 and B4B2 (A, B); TEM of strains R2B1 and B4B2 (C, D).

Fourteen 16S rRNA gene sequences (1386 bp) of isolated strains were obtained and subjected to comparative analysis with the 16S rRNA genes of closely related reference strains. According to the Figure 1, it is evident that these sequences obtained before and after Cu shock load formed distinct clusters in the neighbour-joining phylogenetic tree, referred to as Group I and Group II, respectively. Phylogenetic analysis revealed that both these two groups were within the family Geobacteraceae in the δ-Proteobacteria. The 16S rRNA gene sequences of isolated strains had the closest match to *Enterobacter ludwigii* DSM 16688^T^, ranging from 98.88% to 99.15%. Due to the high similarity among the strains obtained before (after) shock load, two representative strains R2B1 (before Cu shock load) and B4B2 (after Cu shock load) were chosen for further analysis. Strains R2B1 and B4B2 are gram-negative and the SEM and TEM images illustrated that they are 0.5–0.6 µm wide and 0.7–1.8 µm long rod, with a polar flagellum (Figure 2A, B, C, D). The cells occurred in aggregation, and had a few laterally inserted flagella and peritrichous cilium. The fact that these strains can grow both aerobically and anaerobically suggests that the strains are facultative anaerobes. These properties are similar to those of genus *Enterobacter* according to Bergey's Manual of Systematic Bacteriology. However, TEM images indicate that the pili of strain R2B1 are more than those of B4B2 (Figure 2C, D). In addition, when the isolated strains were cultivated on the LB agar plate, strain R2B1 showed a dramatically higher growth rate than that of strain B4B2 under the same cultivation condition.

### Comparison of Cu resistance between *Enterobacter* sp. R2B1 and B4B2

Since *Enterobacter* sp. R2B1 and B4B2 were isolated from a MFC system which was exposed before and after a Cu shock load, the growth of these two strains under a series of different Cu(II) concentrations, ranging from 0 to 400 mg L^−1^, was conducted to investigate the Cu resistance of these two strains (Figure S2). Even though the two isolates both belonged to *Enterobacter* and had high similarity, they exhibited different characteristics of Cu resistance. Figure S2 illustrates that B4B2, which was isolated after Cu shock load, could grow on the LB agar with 200 mg L^−1^ of Cu(II) concentration, while R2B1 could not. In addition, it was found that strain B4B2 grew more rapidly compared with strain R2B1 on the LB agar plate in the presence of Cu(II). These results implied that the Cu shock load promoted the selection of Cu resistant exoelectrogens.

### Biochemical and enzymatic characterization

Biochemical characterization of *Enterobacter* sp. strains R2B1 and B4B2 to use various carbon sources are shown in Table S2. Rezaei et al. [14] reported that *Enterobacter cloacae* FR could produce electricity in an MFC. Therefore, selective carbon sources utilized by *Enterobacter cloacae* FR and the type strain *Enterobacter cloacae* ATCC 13047^T^ were compared with strains R2B1 and B4B2. As shown in Table 1, there were some different carbon utilization patterns among the four strains. Especially, only strain R2B1 oxidized D-Lactose, whereas other strains did not show this capacity. Both strains R2B1 and B4B2 utilized gentiobiose, D-glucose, acetic acid and formic acid, while neither *E. cloacae* FR nor *E. cloacae* ATCC 13047^T^ could use these carbon sources.

**Table 1:** Different carbon sources utilized by *Enterobacter* species.

Carbon sources	*E. cloacae* ATCC 13047^T^	*E. cloacae* FR	Strain R2B1	Strain B4B2
Dextrin	+	+	+	+
Glycogen	w	w	w	w
N-Acetyl-D-glucosamine	+	+	+	+
D-Cellobiose	+	+	+	+
L-Arabinose	+	+	+	+
Gentiobiose	w	w	+	+
D-Glucose	w	w	+	+
D-Lactose	+	+	w	−
Sucrose	+	+	+	+
Acetic acid	w	w	+	+
cis-Acontic acid	+	+	+	+
Citric acid	+	+	+	+
Formic acid	w	w	+	+
Lactic acid	+	+	+	+
Glycerol	+	+	+	+

“w”-weak positive, “+”-Positive, “−”-Negative.

For the API ZYM enzyme assay, nine enzymes were produced by both of the isolates, including phosphatase, leucine aminopeptidase, valine aminopeptidase, cystine aminopeptidase, trypsin, acid phosphatase, β-galactosidase, α-glucosidase and n-acetyl-β-glucosaminidase (Table 2). Different enzyme activities in esterase, lipase and β-glucosidase between the two isolates were observed.

**Table 2:** Enzyme activity of strains R2B1 and B4B2.

ZYM	Strain R2B1	Strain B4B2
Control	−	−
Alkaline phosphatase	+	+
Esterase (C4)	−	+
Esterase lipase (C8)	w	w
Lipase (C14)	w	+
Leucine aminopeptidase	+	+
Valine aminopeptidase	+	+
Cystine aminopeptidase	+	+
Trypsin	+	+
α-chymotrypsin	w	−
Acid phosphatase	+	+
Naphtol-AS-Bl-phosphoamidase	+	w
α-galactosidase	w	−
β-galactosidase	+	+
β-glucuronidase	w	−
α-glucosidase	+	+
β-glucosidase	w	+
N-acetyl-β-glucosaminidase	+	+
α-mannosidase	−	−
α-fucosidase	−	−

“w”-weak positive, “+”-Positive, “−”-Negative.

### Characterization of electrochemical activity

**Figure 3:**
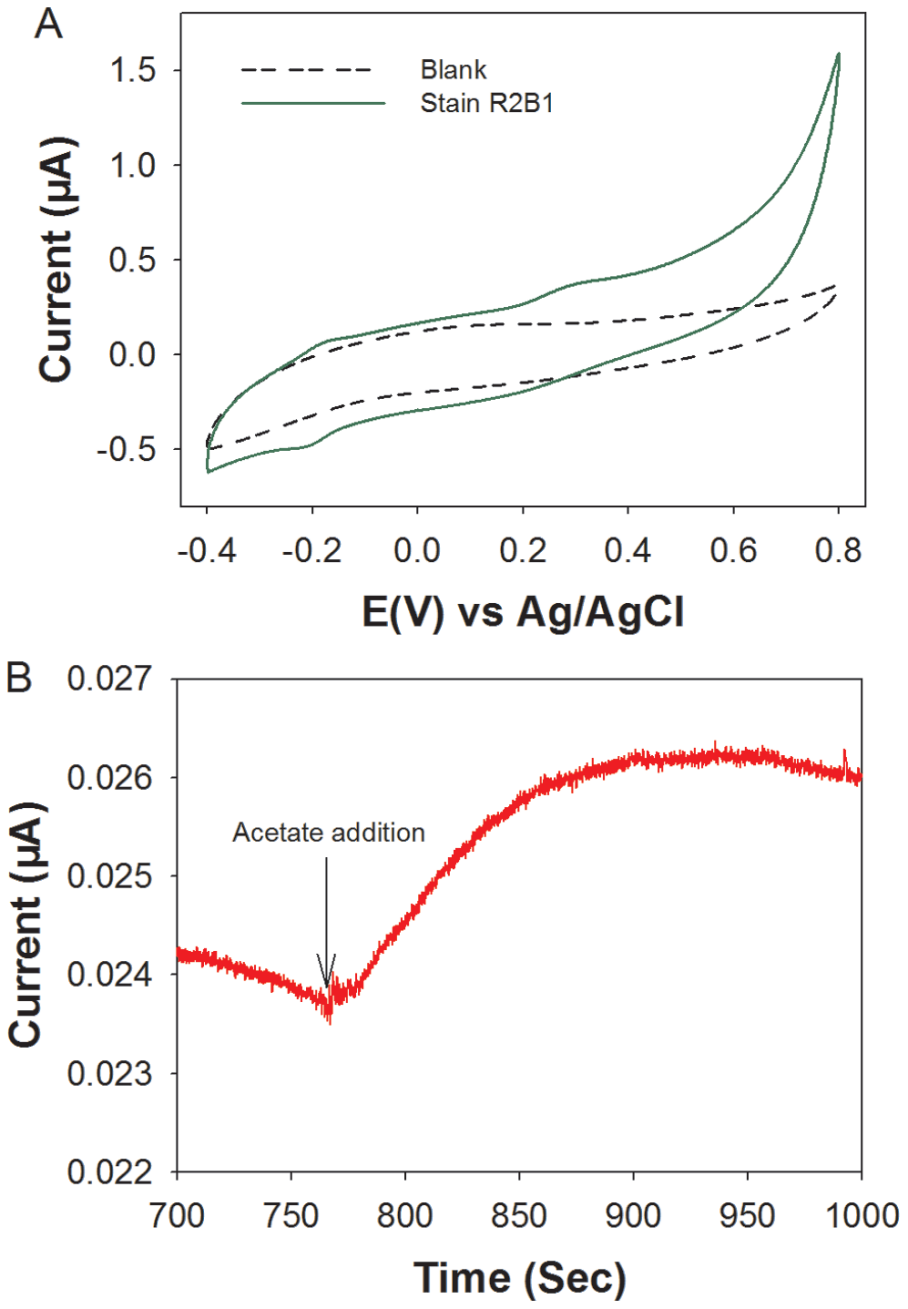
Electrochemical investigation by applying three-electrode set-up. (A) CV scan of strain R2B1 in deoxygenated 0.05 M PBS solution. (B) Changes of current by strain R2B1. Arrows indicate addition of 20 µl acetate (10 mM).

To further investigate the electrochemical characteristics, CV analysis of strain R2B1 on glassy carbon were conducted using a three-electrode system in 50 mM deoxygenated PBS solution. As shown in Figure 3A, no appreciable redox peak was observed without cells. The CV results revealed the presence of redox peaks when the electrode was immobilized with cells. Two oxidation peaks at approximately −0.198 and 0.290 V (vs Ag/AgCl), as well as one reduction peak at around −0.206 V (vs Ag/AgCl) were observed, suggesting that the presence of redox active compounds were involved in extracellular electron transfer by *Enterobacter* sp. strain R2B1. The oxidation peak corresponding to −0.198 V (vs Ag/AgCl) was likely due to the c-type cytochromes [30]–[32], suggesting that the electrochemical activity of strain R2B1 could be related to the plasma membrane protein. Furthermore, the i-t curve demonstrated that there was a rise in current with the addition of acetate, indicating a promotion of electron transfer by adding substrate (Figure 3B).

**Figure 4:**
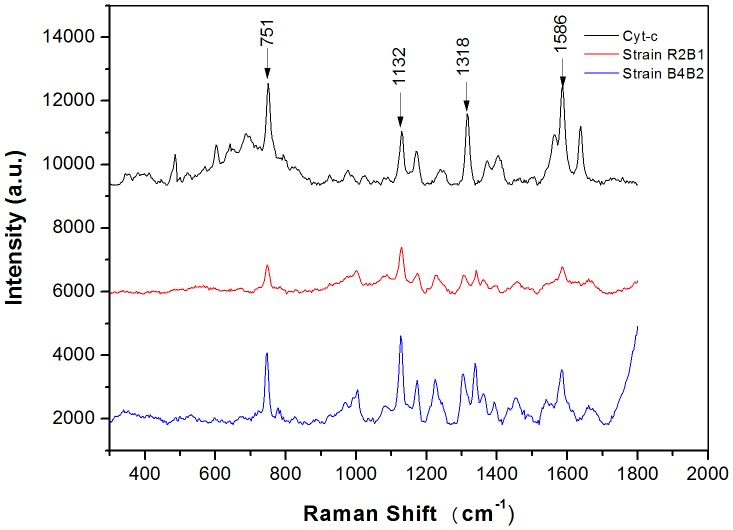
Typical Raman spectra of Cyt-c and isolated pure strains.

Certain proteins, such as cytochromes, are crucial in electron transfer via the physical contact of the bacterial cell membrane. It has been demonstrated that cytochrome *c* are at high densities in electroactive bacteria as they play significant roles in dissimilatory reduction of Fe and Mn oxides [33], electricity production in bioelectrochemical systems [34], and interspecies electron transfer in anaerobic microbial aggregates [35]–[36]. Hence, cytochrome *c* was used as the indicator for the characterization of an electroactive biofilm using Raman microscopy [37]. Cytochrome *c* produced strong, representative and reproducible Raman spectra when excited with a 532 nm wavelength due to the enhancement of the Raman scattering (Raman resonance effect) of the porphyrin ring in visible and ultra-violet bands [37]. Thus, a Raman experiment was conducted to investigate whether the two isolated strains contained cytochrome *c*. According to the results (Figure 4), four typical Raman peaks of cytochrome *c* (751, 1132, 1318, and 1586 cm^−1^) were observed for the two isolated strains, indicating the potential high densities of cytochrome *c* in them. Previous studies reported that cytochrome *c* associated with bacterial outer membrane and conductive pili could be used for direct electron transfer [4]. Interestingly, the isolated bacteria have a large number of pili as shown in the electron microscopic images (Figure 2), and pili of exoelectrogens might serve as microbial nanowires to aid in the electron transfer from the cell surface to the surface of Fe(III) oxides [6]. In addition, a previous study has proposed that in situ oxidation of produced hydrogen might also contribute to the mechanism of electricity generation by *Enterobacter aerogenes* biofilms [38]. Hence, further experiments are required to confirm the exact mechanism which is employed by the isolated bacteria to transfer electron to the surface of insoluble electron acceptors.

### Effect of different substrates on the electricity production

**Figure 5:**
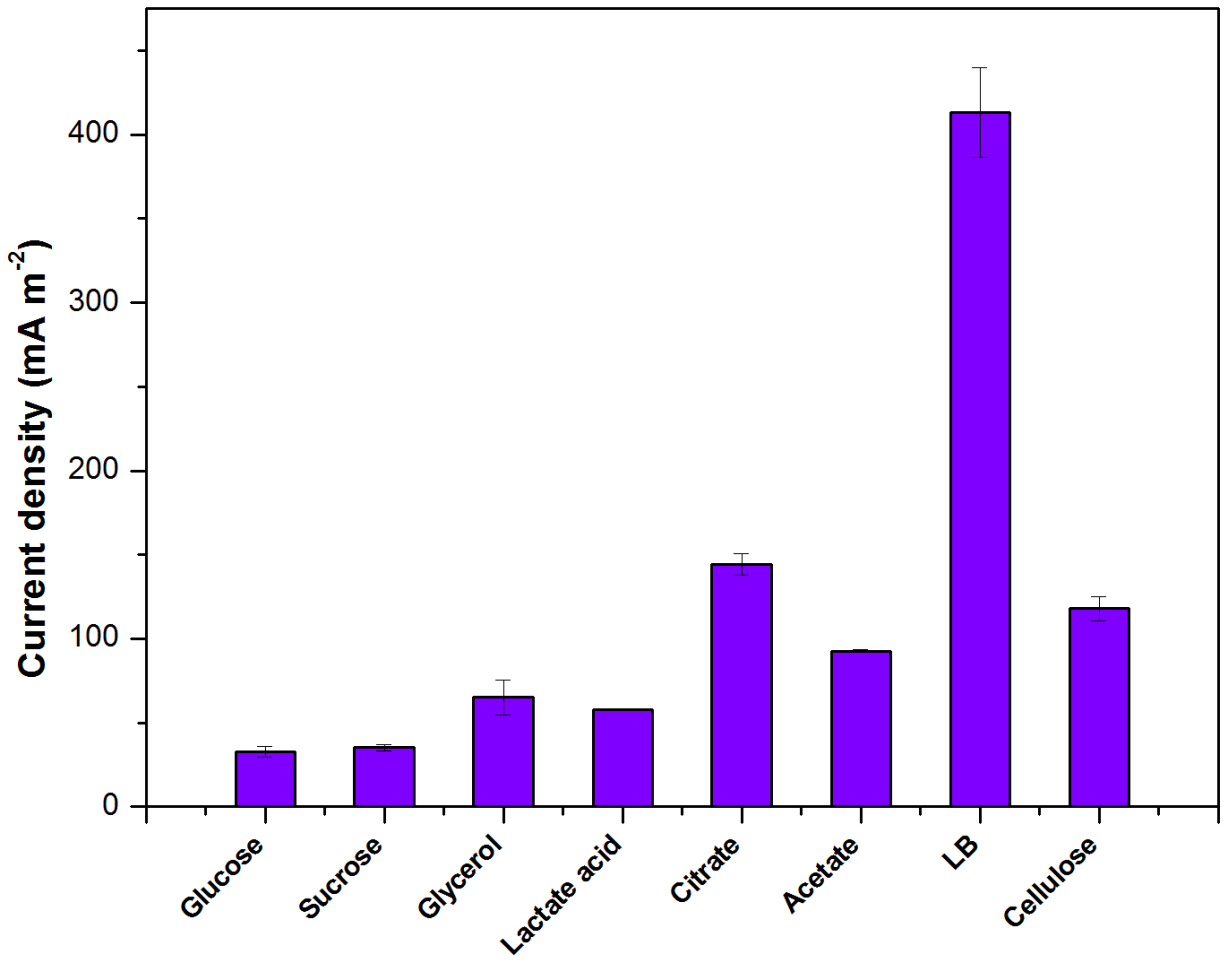
Electricity generation of *Enterobacter* sp. strain R2B1 in a duel chamber MFC with different substrates. The error bar stands for the standard deviation from three or more separate batch experiments.

The isolated strain R2B1 was used to determine its ability to generate current from a variety of substrates, including glucose, lactose, sodium acetate, glycerol and sucrose with an external resistance of 1 kΩ (Figure 5). The LB medium produced the maximum current density of 440 mA·m^−2^ at the fixed resistance, presumably due to higher substrate concentrations from tryptone and yeast extract, followed by citrate, cellulose and acetate with 144±6.3 mA·m^−2^, 118±7.3 mA·m^−2^ and 93±0.9 mA·m^−2^, while the maximum current densities of other substrates were rather low (less than 100 mA·m^−2^). These results illustrated that the strain R2B1 could utilize a wide range of substrates for electricity generation in MFCs but with different power generation abilities. Although strain R2B1 in this study and the *Enterobacter cloacae* ATCC 13047^T^ reported by Razaei [14] are within the genus of *Enterobacter*, they display dissimilarity in terms of electricity production from substrates. For instance, strain R2B1 could oxidize acetate for electricity generation, while *E. cloacae* ATCC 13047^T^ could not.

**Figure 6:**
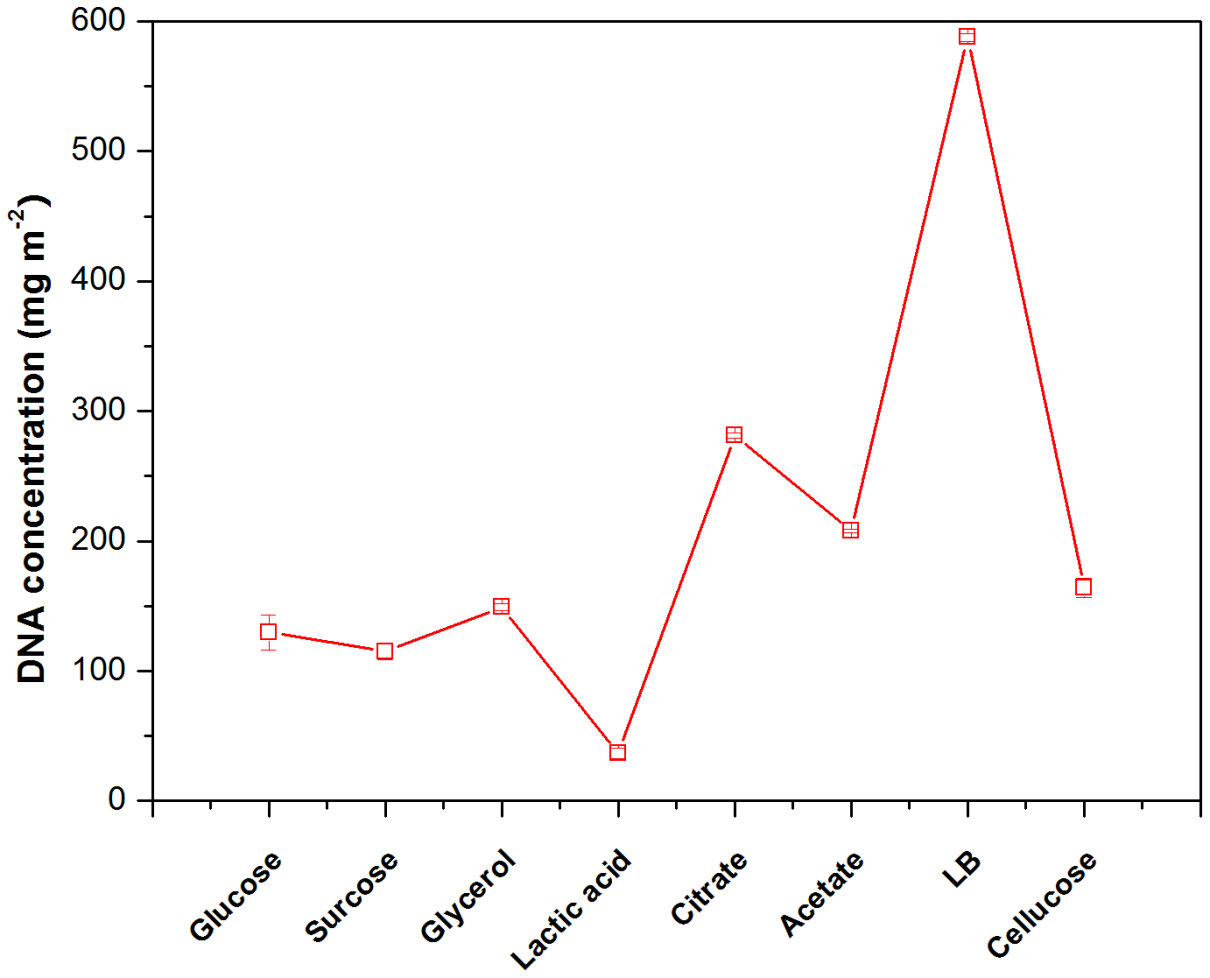
The total DNA on the anode of MFC fed with different substrates.

Since the quantity of cells in the anode biofilm could be one of the crucial factors for the performance of MFC, to estimate the relative bacterial quantity, the total genomic DNA of biofilm sample from MFCs fed with different substrates was extracted (Figure 6). The LB-fed MFCs had the highest number of bacterial cells in the anode biofilm with a DNA contents of approximately 588 mg m^−2^, indicating that LB medium are most suitable for the growth of strain R2B1's anode biofilm. DNA contents of the anode samples from the MFCs fed with citrate, acetate and cellulose were 281, 208 and 164 mg m^−2^, respectively. There was no significant differences among glucose (130 mg m^−2^), sucrose (115 mg m^−2^) and glycerol (150 mg m^−2^) fed MFCs. In general, the bacterial cell density was similar to the trends observed with the current density (Figure 5).

Confocal Raman microscopy was used to analyze the electrochemically active microbial biofilms. We observed that electroactive bacteria were shown to be located preferentially on the surface of the anode carbon felt, probably due to the relatively easy accessibility to substrate (Figure S3). By confocal Raman microscopy analysis, the biofilm thickness on the surface of carbon felt in LB-fed MFC was approximately 13 µm.

## Conclusions

This study reported the isolation of *Enterobacter* strains from an MFC. Raman analysis showed that there was cytochrome *c* in the strains. CV measurements revealed that strain R2B1 could transfer electrons to insoluble extracellular electron acceptors. Strain R2B1 was capable of generating electricity from a wide range of substrates in MFCs, particularly acetate and cellulose. Further experiments indicated that the bacterial cell density correlated with the current density. The findings of this study increase the known diversity of power generating exoelectrogens and the wide range of substrate utilization by strain R2B1 increases the application potential of MFCs in renewable energy generation and waste treatment.

## Supporting Information

Figure S1 **Photos of roll tubes inoculated with samples with ten times dilution.**
(TIF)

Figure S2 **The photographs of the isolated strains cultivated on LB agar plates with different concentrations of copper ions.**
(TIF)

Figure S3 **Images of electroactive biofilms using LB as the carbon source.** Green indicates the electrochemically active biofilm. Black indicates carbon electrode.
(TIF)

Table S1 **Taxa of exoelectrogens.**
(DOC)

Table S2 **Growth on various electron donors of** ***Enterobacter*** **sp. strain R2B1 and B4B2.**
(DOC)
