# Supplementary figures and images for: Characterization of Exoelectrogenic Bacteria Enterobacter Strains Isolated from a Microbial Fuel Cell Exposed to Copper Shock Load
Source: PLoS One. 2014 Nov 20;9(11):e113379. doi: 10.1371/journal.pone.0113379 (PMC4239067; doi:10.1371/journal.pone.0113379)

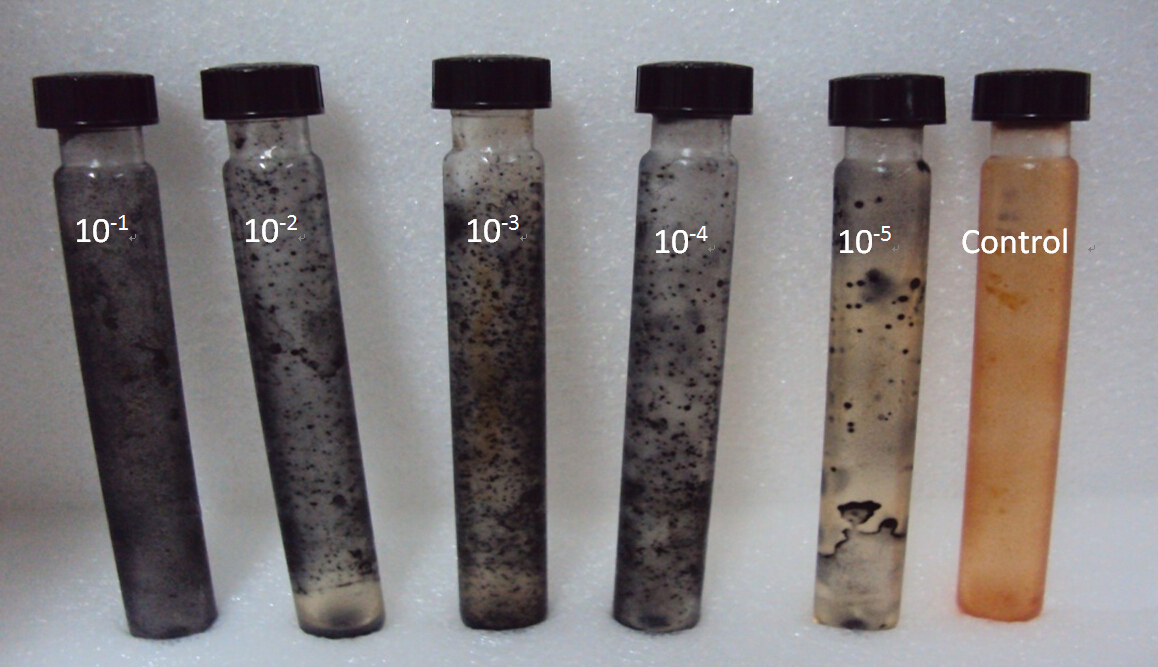

Supplement: Figure S1 — Photos of roll tubes inoculated with samples with ten times dilution. (TIF) [file pone.0113379.s001.tif]

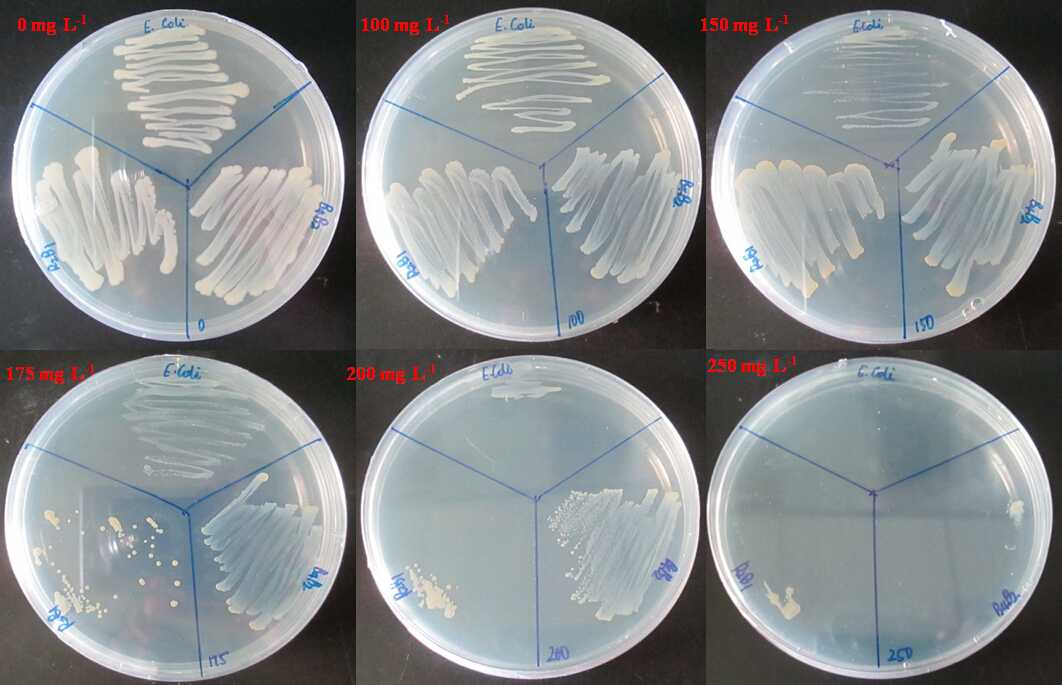

Supplement: Figure S2 — The photographs of the isolated strains cultivated on LB agar plates with different concentrations of copper ions. (TIF) [file pone.0113379.s002.tif]

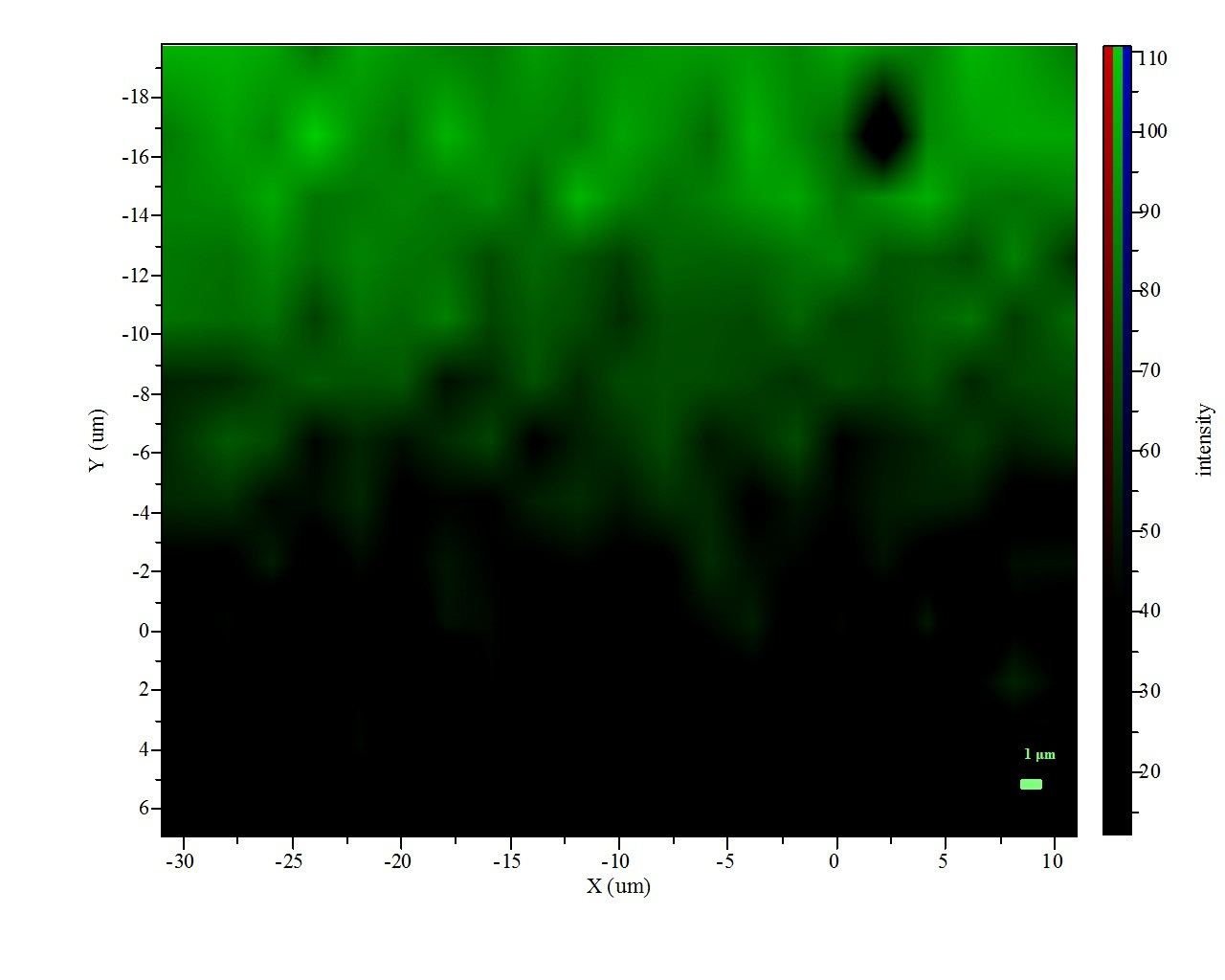

Supplement: Figure S3 — Images of electroactive biofilms using LB as the carbon source. Green indicates the electrochemically active biofilm. Black indicates carbon electrode. (TIF) [file pone.0113379.s003.tif]
