# Supplementary material for: Characterization of Exoelectrogenic Bacteria Enterobacter Strains Isolated from a Microbial Fuel Cell Exposed to Copper Shock Load
Source: PLoS One. 2014 Nov 20;9(11):e113379. doi: 10.1371/journal.pone.0113379 (PMC4239067; doi:10.1371/journal.pone.0113379)
Supplement: Table S1 — Taxa of exoelectrogens. (DOC) [file pone.0113379.s004.doc]

**Table S1.** Taxa of exoelectrogens

| Classifer | Exoelectrogens | Carbon sources | Electrode materials | Current density (mA·m^–2^) | Power density (mW·m^–2^) | Configuration | Columbic efficiency (%) | Ref. |
| --- | --- | --- | --- | --- | --- | --- | --- | --- |
| *α*-proteobacteria | *Rhodopseudomonas palustris* DX-1 | Acetate | Graphite brush | 9900 | 2720 | Single-chamber | — |  |
|  | *Ochrobactrum anthropi* YZ-1 | Acetate | Carbon cloth | 708 | 89 | U shape | > 80 |  |
|  | *Acidiphilium sp*. strain 3.2 Sup 5 | Glucose | — | 3000 | — | — | — |  |
| *β*-proteobacteria | *Rhodoferax ferrireducens* strain T118 | Glucose, xylose, sucrose, maltose | Plain graphite | 74 | 17.67 | Two chamber | 81 |  |
| *γ*-proteobacteria | *Shewanella oneidensis* strain MR-1 | — | Graphite felt | 18 | — | Two chamber | — |  |
|  | *Shewanella oneidensis* DSP10 | lactate | glassy carbon | 100 | 24 | — | < 10 |  |
|  | *Shewanella. putrefaciens* | Acetate, pyruvate, glucose, lactate | Graphite | 312.5 | 10.2 | Single-chamber | 20 |  |
|  | *Enterobacter cloacae* | Cellulose, sucrose, glycerol | Carbon cloth | 493 8 | 4.9 | U tube | 26 |  |
|  | *Citrobacter sp*. SX-1 | Citrate, glycerol, sucrose | Carbon cloth | 58 | 88.1 | Single-chamber | — |  |
|  | *Pseudomonas aeruginosa* strain KRP1 | Glucose | Graphite sheets | — | 28.4 | Two-chamber | — |  |
|  | *Klebsiella pneumonia* strain L17 | Glucose | Carbon felt | 0.12 | 409.71 | Two-chamber | — |  |
|  | *Aeromonas sp.* strain ISO2-3 | Glucose | Graphite | — | 800 | Two-chamber | — |  |
|  | *Aeromonas hydrophila* PA3 | Yeast extract | — | — | — | — | — |  |
| *δ*-proteobacteria | *Geobacter sulfurreducens* | Acetate | Graphite | 4560 | 1880 | Two-chamber | 100 |  |
|  | *Geobacter sulfurreducens* | Acetate | Graphite | 1143 | 15 | Two-chamber | 96.8 |  |
|  | *Geobacter sulfurreducens* strain PCA | Acetate | Graphite | 65 | — | Two-chamber | — |  |
|  | *Geobacter metallireducens* | Acetate | Carbon paper | — | 40 | Two-chamber | — |  |
|  | *Geobacter electrodiphilus* | Acetae, malic, fumaric and citric acid | Graphite | 121.43 | — | Two-chamber | 96.30 |  |
|  | *Desulfobulbus propionicus* | Lactic, pyruvic, propionic acid | Graphite | 28.35 | — | Two-chamber | 26.4 |  |
| *ε*-proteobacteria | *Arcobacter butzleri* strain ED-1 | Acetate | Graphite felt | — | 296 mW L^–1^ | — | — |  |
| Firmicutes | *Clostridium butyricum* EG3 | Glucose | Graphite felt | — | — | — |  |  |
|  | *Thermincola* sp. strain JR | Acetate | Graphite | — | 37 | Two-chamber | — |  |
| Acidobacteria | *Geothrix fermentans* | Acetate, propionate, malate, lactate, or succinate | Graphite | 50 | — | Two-chamber | 97 |  |
| Actinobacteria | *Propionibacterium freudenreichii* ET-3 | Glucose | Carbon felt | — | — | Two-chamber | — |  |

**References**

1. Xing D, Zuo Y, Cheng S, Regan JM and Logan BE (2008) Electricity generation by *Rhodopseudomonas palustris* DX-1. Environ Sci Technol 42: 4146-4151.
2. Zuo Y, Xing D, Regan JM and Logan BE (2008) Isolation of the exoelectrogenic bacterium Ochrobactrum anthropi YZ-1 by using a U-tube microbial fuel cell. Appl Environ Microb 74: 3130-3137.
3. Malki M, De Lacey AL, Rodríguez N, Amils R and Fernandez VM (2008) Preferential use of an anode as an electron acceptor by an acidophilic bacterium in the presence of oxygen. Appl Environ Microb 74: 4472-4476.
4. Chaudhuri SK and Lovley DR (2003) Electricity generation by direct oxidation of glucose in mediatorless microbial fuel cells. NAT BIOTECHNOL 21: 1229-1232.
5. El-Naggar MY, Gorby YA, Xia W and Nealson KH (2008) The molecular density of states in bacterial nanowires. Biophys J 95: L10-L12.
6. Ringeisen BR, Henderson E, Wu PK, Pietron J, Ray R, et al. (2006) High power density from a miniature microbial fuel cell using Shewanella oneidensis DSP10. Environ Sci Technol 40: 2629-2634.
7. Park D and Zeikus J (2002) Impact of electrode composition on electricity generation in a single-compartment fuel cell using Shewanella putrefaciens. Appl Microbiol Biot 59: 58-61.
8. Rezaei F, Xing D, Wagner R, Regan JM, Richard TL, et al. (2009) Simultaneous cellulose degradation and electricity production by Enterobacter cloacae in a microbial fuel cell. Appl Environ Microb 75: 3673-3678.
9. Xu S and Liu H (2011) New exoelectrogen *Citrobacter* sp. SX‐1 isolated from a microbial fuel cell. J Appl Microbiol 111: 1108-1115.
10. Rabaey K, Boon N, Siciliano SD, Verhaege M and Verstraete W (2004) Biofuel cells select for microbial consortia that self-mediate electron transfer. Appl Environ Microb 70: 5373-5382.
11. Zhang L, Zhou S, Zhuang L, Li W, Zhang J, et al. (2008) Microbial fuel cell based on *Klebsiella pneumoniae* biofilm. Electrochem Commun 10: 1641-1643.
12. Chung K and Okabe S (2009) Characterization of electrochemical activity of a strain ISO2‐3 phylogenetically related to Aeromonas sp. isolated from a glucose‐fed microbial fuel cell. Biotechnol Bioeng 104: 901-910.
13. Pham CA, Jung SJ, Phung NT, Lee J, Chang IS, et al. (2003) A novel electrochemically active and Fe (III)‐reducing bacterium phylogenetically related to Aeromonas hydrophila, isolated from a microbial fuel cell. FEMS Microbiol Lett 223: 129-134.
14. Nevin K, Richter H, Covalla S, Johnson J, Woodard T, et al. (2008) Power output and columbic efficiencies from biofilms of Geobacter sulfurreducens comparable to mixed community microbial fuel cells. Environ Microbiol 10: 2505-2514.
15. Bretschger O, Obraztsova A, Sturm CA, Chang IS, Gorby YA, et al. (2007) Current production and metal oxide reduction by Shewanella oneidensis MR-1 wild type and mutants. Appl Environ Microb 73: 7003-7012.
16. Bond DR and Lovley DR (2003) Electricity production by *Geobacter sulfurreducens* attached to electrodes. Appl Environ Microb 69: 1548-1555.
17. Min B, Cheng S and Logan BE (2005) Electricity generation using membrane and salt bridge microbial fuel cells. Water Res 39: 1675-1686.
18. Holmes DE, Nicoll JS, Bond DR and Lovley DR (2004) Potential role of a novel psychrotolerant member of the family Geobacteraceae, Geopsychrobacter electrodiphilus gen. nov., sp. nov., in electricity production by a marine sediment fuel cell. Appl Environ Microb 70: 6023-6030.
19. Holmes DE, Bond DR and Lovley DR (2004) Electron transfer by Desulfobulbus propionicus to Fe (III) and graphite electrodes. Appl Environ Microb 70: 1234-1237.
20. Fedorovich V, Knighton MC, Pagaling E, Ward FB, Free A, et al. (2009) Novel electrochemically active bacterium phylogenetically related to Arcobacter butzleri, isolated from a microbial fuel cell. Appl Environ Microb 75: 7326-7334.
21. Park HS, Kim BH, Kim HS, Kim HJ, Kim GT, et al. (2001) A Novel Electrochemically Active and Fe (III)-reducing Bacterium Phylogenetically Related to *Clostridium butyricumi* Isolated from a Microbial Fuel Cell. Anaerobe 7: 297-306.
22. Wrighton KC, Agbo P, Warnecke F, Weber KA, Brodie EL, et al. (2008) A novel ecological role of the Firmicutes identified in thermophilic microbial fuel cells. The ISME Journal 2: 1146-1156.
23. Bond DR and Lovley DR (2005) Evidence for involvement of an electron shuttle in electricity generation by Geothrix fermentans. Appl Environ Microb 71: 2186-2189.
24. Wang YF, Masuda M, Tsujimura S and Kano K (2008) Electrochemical regulation of the end‐product profile in Propionibacterium freudenreichii ET‐3 with an endogenous mediator. Biotechnol Bioeng 101: 579-586.
