# Supplementary material for: Characterization of Exoelectrogenic Bacteria Enterobacter Strains Isolated from a Microbial Fuel Cell Exposed to Copper Shock Load
Source: PLoS One. 2014 Nov 20;9(11):e113379. doi: 10.1371/journal.pone.0113379 (PMC4239067; doi:10.1371/journal.pone.0113379)
Supplement: Table S2 — Growth on various electron donors of Enterobacter sp. strain R2B1 and B4B2. (DOC) [file pone.0113379.s005.doc]

**Table S2.** Growth on various electron donors of *Enterobacter* sp. strain R2B1 and B4B2

| Carbon source | Strain R2B1 | Strain B4B2 | Carbon source | Strain R2B1 | Strain B4B2 | |
| --- | --- | --- | --- | --- | --- | --- |
| Water | - | - | p-Hydroxy phenylacetic acid | + | + |  |
| α-cyclodextrin | - | - | Itaconic acid | - | - |  |
| Dextrin | + | + | α-Keto butyric acid | - | w |  |
| Glycogen | W | W | α-Keto glutaric acid | - | - |  |
| Tween 40 | W | W | α-Keto valeric acid | - | - |  |
| Tween 80 | + | + | DL-lactic acid | + | + |  |
| N-acetyl-D-galactosamine | - | + | Malonic acid | + | - |  |
| N-acetyl-D-glucosamine | + | + | Propionic acid | - | - |  |
| Adonitol | - | - | Quinic acid | - | - |  |
| L-arabinose | + | + | D-saccharic acid | + | - |  |
| D-arabitol | - | - | Sebacic acid | - | - |  |
| D-cellobiose | + | + | Succinic acid | + | + |  |
| i-erythritol | - | - | Bromo succinic acid | + | + |  |
| D-fructose | + | + | Succinamic acid | - | w |  |
| L-fucose | - | + | Glucuronamide | w | + |  |
| D-galactose | + | + | L-alaninamide | + | + |  |
| Gentiobiose | + | + | D-alanine | + | + |  |
| α-D-glucose | + | + | L-alanine | + | + |  |
| m-inositol | + | + | L-alanyl-glycine | + | + |  |
| α-D-lactose | w | - | L-asparagine | + | + |  |
| Lactulose | - | - | L-aspartic acid | + | + |  |
| Maltose | + | + | L-glutamic acid | + | + |  |
| D-mannitol | + | + | glycyl-L-aspartic acid | + | + |  |
| D-mannose | + | + | glycyl-L-glutamic acid | + | + |  |
| D-melibiose | + | - | L-histidine | + | + |  |
| β-methyl-D-glucoside | + | + | Hydroxy-L-proline | - | + |  |
| D-psicose | + | + | L-leucine | - | - |  |
| D-raffinose | + | - | L-ornithine | + | w |  |
| L-rhamnose | + | - | L-phenylalanine | + | + |  |
| D-sorbitol | + | + | L-proline | + | + |  |
| Sucrose | + | + | L-pyroglutamic acid | - | - |  |
| D-trehalose | + | + | D-serine | + | + |  |
| Turanose | + | + | L-serine | + | + |  |
| Xylitol | - | - | L-threonine | + | + |  |
| Methyl pyruvate | + | + | DL-carnitine | - | - |  |
| mono-methyl-succinate | w | + | γ-amino butyric acid | - | - |  |
| Acetic acid | + | + | Urocanic acid | + | - |  |
| Cis-aconitic acid | + | + | Inosine | + | + |  |
| Citric acid | + | + | Uridine | + | + |  |
| Formic acid | + | + | Thymidine | + | + |  |
| D-galactonic acid lactone | + | - | Phenyethylamine | - | - |  |
| D-galacturonic acid | + | + | Putrescine | + | - |  |
| D-gluconic acid | + | + | 2-aminoethanol | - | - |  |
| D-glucosaminic acid | - | - | 23-butanediol | - | - |  |
| D-glucuronic acid | + | + | Glycerol | + | + |  |
| α-Hydroxy butyric acid | w | - | DL-α-glycerol phosphate | + | - |  |
| β-Hydroxy butyric acid | + | + | Glucose-1-phosphate | + | + |  |
| γ-Hydroxy butyric acid | - | - | Glucose-6-phosphate | + | + |  |

“w”-weak positive，“+”-Positive，“–”-Negative
